# Tanshinone IIA alleviates atherosclerosis in LDLR^−/−^ mice by regulating efferocytosis of macrophages

**DOI:** 10.3389/fphar.2023.1233709

**Published:** 2023-10-11

**Authors:** Jiarou Wang, Yifan Zhang, Xiaoteng Feng, Min Du, Sijin Li, Xindi Chang, Ping Liu

**Affiliations:** Longhua Hospital, Shanghai University of Traditional Chinese Medicine, Shanghai, China

**Keywords:** tanshinone IIA, efferocytosis, macrophage, lipid metabolism, atherosclerosis

## Abstract

**Background:** Tanshinone IIA (TIIA) is the major lipid-soluble active ingredient of the traditional Chinese medicine *Salvia miltiorrhiza*, which slows down atherosclerosis (AS). However, it remains unclear whether TIIA has the potential to enhance macrophage efferocytosis and thereby improve atherosclerosis.

**Objective:** The focus of this examination was to determine if TIIA could reduce lipid accumulation and treat AS by enhancing efferocytosis.

**Methods:** Firstly, we conducted *in vivo* experiments using LDLR knockout (LDLR^−/−^) mice for a period of 24 weeks, using histopathological staining, immunofluorescence and Western blot experiments to validate from the efficacy and mechanism parts, respectively; in addition, we utilized cells to validate our study again *in vitro*. The specific experimental design scheme is as follows: *In vivo*, Western diet-fed LDLR^−/−^ mice for 12 weeks were constructed as an AS model, and normal diet-fed LDLR^−/−^ mice were taken as a blank control group. The TIIA group and positive control group (atorvastatin, ATO) were intervened for 12 weeks by intraperitoneal injection (15 mg/kg/d) and gavage (1.3 mg/kg/d), respectively. *In vitro*, RAW264.7 cells were cultured with ox-LDL (50 ug/mL) or ox-LDL (50 ug/mL) + TIIA (20 uM/L or 40 uM/L). Pathological changes in aortic plaques and foam cell formation in RAW264.7 cells were evaluated using Masson and Oil Red O staining, respectively. Biochemical methods were used to detect lipid levels in mice. The immunofluorescence assay was performed to detect apoptotic cells and efferocytosis-related signal expression at the plaques. RT-qPCR and Western blot were carried out to observe the trend change of efferocytosis-related molecules in both mouse aorta and RAW264.7 cells. We also used the neutral red assay to assess RAW264.7 cells’ phagocytic capacity.

**Results:** Compared with the model group, TIIA decreased serum TC, TG, and LDL-C levels (*p* < 0.01), reduced the relative lumen area of murine aortic lipid-rich plaques (*p* < 0.01), enhanced the stability of murine aortic plaques (*p* < 0.01), reduced ox-LDL-induced lipid build-up in RAW264.7 cells (*p* < 0.01), and upregulated efferocytosis-related molecules expression and enhance the efferocytosis rate of ox-LDL-induced RAW264.7 cells.

**Conclusion:** TIIA might reduce lipid accumulation by enhancing the efferocytosis of macrophages and thus treat AS.

## 1 Introduction

Cardiovascular disease (CVD) is one of the leading causes of death worldwide, and its mortality rates have persisted at high levels for numerous years (Tsao et al., 2022). The pathological basis of many cardiovascular diseases is atherosclerosis (AS), which has a complex pathogenesis mainly involving theories of immune inflammation and lipid metabolism (Björkegren and Lusis, 2022). The neurological and muscular side effects of statin lipid-lowering drugs and the risk of post-surgical complications have greatly limited the clinical application of the two existing mainstream treatment strategies (Pierno and Musumeci, 2023). Clinical studies have shown that invasive treatments may not always be feasible and can increase the risk of complications such as thrombosis or bleeding within 6 months after surgery for patients (Sherwood et al., 2016; Badjatiya and Rao, 2019). To make matters worse, the incidence of AS has been trending toward a younger age in recent years (Libby, 2021). However, compared to other diseases, developing new drugs for AS is significantly lagging (Brown and Wobst, 2021). This requires us to find new effective treatment strategies as soon as possible.

Various immune cells are involved in the pathological process of AS. Macrophages, as core members of intrinsic immunity, are AS’s most crucial cell type (Chen et al., 2022; Doddapattar et al., 2022). Macrophage efferocytosis refers to the process by which macrophages phagocytically degrade apoptotic cells (Jia et al., 2022). Effective efferocytosis can remove necrotic and dead cells from the lesion and reduce tissue damage and AS (Gerlach et al., 2021). Impaired efferocytosis may further accelerate AS progression (Kasikara et al., 2021). Thus, efficient efferocytosis is closely linked to AS, and targeting the efferocytosis of macrophages may be an effective AS treatment strategy.

Traditional Chinese medicine (TCM) and its active ingredients are gradually gaining advantages in clinical treatment because of its economic, safe, and efficient features. In recent decades, the practical application of TCM in the clinical treatment of cardiovascular-related diseases has brought new hope for treating AS (Hao et al., 2017). TIIA is the main active ingredient of *Salvia miltiorrhiza*, which has been shown to have therapeutic effects on AS (Wang et al., 2020; Wen et al., 2020). TIIA can also treat neurological diseases such as Parkinson’s by exerting anti-inflammatory and antioxidant effects. However, it is unclear whether TIIA could improve AS via modulating efferocytosis. This investigation aimed to explore whether TIIA could reduce lipid accumulation and enhance efferocytosis to treat AS.

AS this study focuses on the therapeutic effects and mechanisms of Tanshinone IIA (TIIA) on AS, it is especially critical to select an appropriate animal model of AS for animal-level studies. In humans, AS is formed mainly by abnormal accumulation of lipids such as cholesterol due to the long-term Western diet. The establishment of animals for AS is reported to have undergone a long process, and several researchers have explored a great deal from animals such as zebrafish, rabbits, and guinea pigs after continuous efforts, but all of these models have limitations in application. Mice are widely popular due to rapid reproduction, availability of genetic manipulation, and reasonable time to induce atherosclerotic lesions. However, wild-type mice are not susceptible to plaque formation because of their low levels of apolipoprotein-containing lipoproteins, which are quite resistant to the development of AS. Fortunately, with the development of gene editing technology, scientists worked out LDLR knockout (LDLR^−/−^) mice in 1993. Compared with wild-type mice, LDLR^−/−^ mice are more prone to plaque formation induced by a high-fat diet (Western diet), and the process of development is similar to the process of formation of AS in humans caused by a prolonged Western diet (Gisterå et al., 2022), since then LDLR^−/−^ mice have been widely used as AS model mice (Simion et al., 2020; Doddapattar et al., 2022; Jin et al., 2022; Manta et al., 2022). Therefore, in the present study, LDLR^−/−^ mice were selected to establish AS model mice induced by Western diet to further investigate the mechanism of action of TIIA in the treatment of AS.

## 2 Materials and methods

### 2.1 Reagents

Tanshinone IIA was bought from Mansite Biological Company (19032104, HPLC≥98%, Chengdu, China). Western diet (21% fat and 0.15% cholesterol) was produced by SYSE Biotechnology (Guangzhou, China). The oil red O (ORO) staining kit and Masson staining kit were from the Jiancheng Bioengineering Institute of Nanjing (Nanjing, China). 4′, 6-diamindino-2-phenylindole (DAPI), Triton X-100, Radio ImmunoPrecipitation Assay (RIPA), and BCA protein assay kit were from Beyotime Biotechnology (Shanghai, China). Primers for β-ACTIN, MFGE8, CX3CR1, MERTK, AXL, and TYRO3 were obtained by Sangon Biotech Co.

### 2.2 Animal husbandry and management

The animal study was approved by the Ethics Committee of Longhua Hospital Affiliated with Shanghai University of Traditional Chinese Medicine (No. 2019-N002, shown in Supplementary Material). All mice (32 LDLR^−/−^ mice, 6 weeks old) were purchased from Gempharmacy Co., Ltd (Nanjing, CN, https://www.gempharmatech.com/). Mice are housed in SPF class animal rooms at Longhua Hospital. The model (MOD), TIIA, and positive control (atorvastatin, ATO) groups were created by randomly assigning the LDLR^−/−^ mice to each group. To create an atherosclerotic mouse model, all LDLR^−/−^ mice were given a Western diet for the initial 12 weeks of the experiment. We additionally selected 8 wild-type mice of the same age as the control group, and the experiment was performed on a normal diet throughout.

During the 13–24 weeks of the experiment, the intervention was administered daily at regular intervals to the model group (intraperitoneal injection, saline 2 mL/d), the TIIA group (intraperitoneal injection, 15 mg/kg/d), and the ATO group (gavage, 1.3 mg/kg/d). After 12 weeks of continuous intervention, all mice were fasted for 8 h. Under anesthesia, blood samples were retained from each group of mice using the posterior eye vein from the blood sampling method. The abdominal and thoracic cavities of the mice were then dissected and the heart and aortic tissues were carefully stripped with forceps. The upper 2/3 of the heart tissues were fixed in 4% paraformaldehyde. After being fixed in paraformaldehyde, mouse heart specimens were subsequently dehydrated by transferring them into solutions of 10%, 20%, and 30% sucrose water. Finally, the specimens were embedded in OCT gel. The frozen microtome was used to slice the embedded mouse heart tissue, and a thickness of 50 μm was selected for the first section. At the same time, the frozen sections were observed under the microscope. When we saw a Valve, it indicated that the aortic sinus had been cut. At this time, we used a section with a thickness of 10 μm and finally selected the most ideal section for pathological staining.

### 2.3 Cell culture

RAW264.7 cells were procured from the National Collection of Authenticated Cell Cultures located in Shanghai, China. The cell culture medium used was DMEM (SH30022.01, HyClone, United States) complete medium, which contained 1% penicillin/streptomycin (SV30010, HyClone, United States) and 5% fetal bovine serum (FBS, 10099141C, Gibco, United States). The cells were stored in a Thermo, USA-made incubator that was maintained at 37°C and contained 5% CO_2_. To induce the *in vitro* model, 50 μg/mL of oxidized LDL (ox-LDL, Yeasen, China) was used.

### 2.4 Histopathological analysis

ORO staining: RAW264.7 cells were plated in a 24-well plate at 1 × 10^5^ cells per well for 24 h. The next day, the medium was changed, and ox-LDL (50 ug/mL) or ox-LDL (50 ug/mL) + TIIA (20 uM/L or 40 uM/L) was added to induce 24 h. After discarding the medium, three PBS washes, each lasting 5 min, were performed on the cells. A solution of 4% paraformaldehyde was used to fix RAW264.7 cells and mouse aortic sinus frozen sections for 15 min. Then all samples were washed (Raw264.7 cells and frozen sections) three times with PBS for 5 min each. ORO staining solution was diluted two times with sterile water. The samples were stained with diluted ORO stain for 1.5 h at 37°C. Then we washed the sample in the same way. The nuclei were stained for 2 min with the hematoxylin staining solution. Mouse aortic sinus frozen sections were stained according to the requirements of the Masson trichrome staining kit.

### 2.5 Measurement of blood lipid index in mice

The supernatant from the mouse blood samples was transferred to a fresh centrifuge tube after being spun at 4°C for 20 min at 2,000 rpm. We double-diluted the serum with 1xPBS. Finally, TC, TG, and LDL were measured using a fully automated biochemical analyzer (Beckman Coulter DXC700au, US).

### 2.6 Immunofluorescence

RAW264.7 cells and mouse aortic sinus frozen sections were closed with 0.2% BSA-PBST solution for 30 min. Anti-Caspase3, Anti-CX3CR1, 1:100 dilutions, and incubated overnight at 4°C. The next day, The medium was discarded, and PBS was washed 2 times for 5 min each time. The related species’ secondary antibody was incubated at 20°C–25°C for 1 hour. The DAPI dilution was applied to stain the nuclei. Figures were taken under the fluorescent inverted microscope for subsequent quantitative analysis.

### 2.7 Reverse transcription-quantitative polymerase chain reaction (RT-qPCR)

RAW264.7 Cells and mouse aortic total RNA were extracted as described in the Total RNA Extraction Kit (EZB-RN4, EZBioscience, US). Total RNA was then reverse transcribed into cDNA as a template described in the Reverse Transcription Kit (RR037A, TAKARA, CN). Assay samples containing primers for the target genes were configured according to the TB Green^®^ Premix Ex Taq™ kit (RR420A, TAKARA, CN). In Table 1, the primer sequences are displayed. Parameters of the Step One Plus7500RealTimePCR instrument (Applied Biosystems, US) were set. The experiment utilized the control group as the control group and β-Actin as the internal reference.

**TABLE 1:** List of primers for RT-qPCR analysis.

Gene	Oligonucleotide sequence	
β-Actin	Forward	5′-GAG​ACC​TTC​AAC​ACC​CCA​GC- 3′
	Reverse	5′-ATG​TCA​CGC​ACG​ATT​TCC​C- 3′
MFGE8	Forward	5′-TGA​CTT​TGG​ACA​CAC​AGC​GT- 3′
	Reverse	5′-GGG​AGG​CTA​GGT​TGT​TGG​AAA- 3′
CX3CR1	Forward	5′-GAG​TAT​GAC​GAT​TCT​GCT​GAG​G- 3′
	Reverse	5′-CAG​ACC​GAA​CGT​GAA​GAC​GAG- 3′
TYRO3	Forward	5′- ACT​GGC​TTC​TCT​GCT​GCT​C- 3′
	Reverse	5′- AGC​ATC​AGA​CCG​TTC​CAC​TG - 3′
MERTK	Forward	5′-AGT​TTG​GGA​CGT​TGG​TGG​AT- 3′
	Reverse	5′-GGA​CAC​CGT​CAG​TCC​TTT​GT- 3′
AXL	Forward	5′- GAGCCAACCGTGGAAAGA - 3′
	Reverse	5′- AGG​CCA​CCT​TAT​GCC​GAT​CTA- 3′

### 2.8 Western blot

The mouse aorta was crushed by ultrasonic vibrator, and the cells were collected by cell scraping. The samples were lysed by radioimmunoprecipitation assay (RIPA). The lysate was placed on ice for 30 min. To completely lyse the sample, mix the lysate upside down every 10 min. Centrifuge at 4°C, 15,000 rpm for 15 min to acquire protein supernatant. The protein concentration was calculated according to the bicinchoninic acid (BCA) kit. Set a protein loading volume of 20 ug and a loading volume of 15 μL/well. According to the protein concentration, the corresponding RIPA and bromophenol blue volumes were added. Protein samples were separated at 60–90 V for about 2 h. Then the samples were electrotransferred to a PVDF membrane using 350 mA and 1-h duration, and the PVDF membrane was soaked in QuickBlock™ Western sealing fluid (P0252-100 mL, Beyotime Biotechnology) for 30 min. Immerse the membrane in an antibody incubation cassette containing the diluted primary antibody and store the antibody incubation cassette at 4°C overnight. Primary antibodies against MFGE8, CX3CR1, MERTK, AXL, TYRO3, and GAPDH from Cell Signaling Technology Inc. (Beverly, Massachusetts, United States). The next day Tris-buffered saline with Tween-20 (TBST) is washed three times for 15 min each time. The secondary antibody of the related species was incubated for 1 h at room temperature. Development by substrate chemiluminescence. The images were retained for gray value analysis.

### 2.9 Neutral red uptake assay

To examine the impact of TIIA on the efferocytosis rate of RAW264.7 cells, we conducted the Neutral Red Uptake Assay. RAW264.7 cells were plated in a 96-well plate at 1 × 10^4^ cells per well for 24 h. The next day, the medium was changed, and ox-LDL (50 ug/mL) or ox-LDL (50 ug/mL) + TIIA (20 uM/L or 40 uM/L) was added to induce the cells for 24 h. The medium was discarded, and PBS cells were washed. Add 200 μL DMEM complete medium +20 μL neutral red staining solution to each well and incubate in a cell incubator containing 5% CO_2_ at 37°C for 2 h. Discard the medium and wash the cells with PBS. Add 200 μL Neutral Red assay lysate to each well and lyse at room temperature for 10 min in a shaker. The OD value was measured at 540 nm by an enzyme marker.

### 2.10 Statistical analysis

GraphPad Prism 9.0 was used for statistical analysis. The variables data appearing in the experiments were expressed as mean ± standard deviation (SD). Multiple groups of data that conformed to normality were compared using one-way ANOVA with Bonferroni correction, with statistical significance set at *p <* 0.05.

## 3 Results

### 3.1 TIIA inhibits the progression of aortic plaque in mice

**FIGURE 1:**
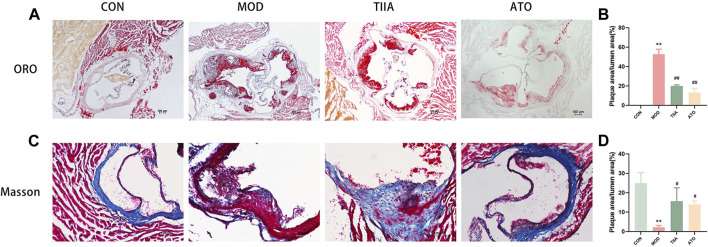
Pathological changes of the aortic sinus in the LDLR^−/−^ mice (n = 3). **(A,B)** Representative images of ORO staining for each group of mouse aortic sinus, and the plaque area of the whole aorta was quantitated. **(C,D)** Representative images of Masson’s staining for each group of mouse aortic sinus and the collagen contents of the whole aorta were quantitated. CON, MOD, ATO, and TIIA represent the control group, model group, atorvastatin group, and tanshinone IIA group, respectively. **p < 0.01 vs. CON; #*p* < 0.05, ##*p* < 0.01 vs. MOD.

To investigate the impact of TIIA on plaques in mice, we employed ORO staining to examine the pathological changes of lipid-rich plaques at the aortic sinus in each group of mice. Our study found that the control group did not have any plaque formation. Conversely, the plaques in the MOD were easily observed (*p* < 0.01). After TIIA or ATO treatment, the relative luminal area of lipid-rich plaques in mice was significantly reduced compared to the model group (Figures 1A, B, *p* < 0.01). The thickness of the fibrous cap at the plaque is one of the essential indicators for detecting plaque stability (Virmani et al., 2006). Therefore, we used Masson staining to observe the collagen fiber content at the plaque. The plaques of mice in the model group had the lowest collagen fiber content. (*p* < 0.01 or *p* < 0.05); The group TIIA or ATO’s aortic plaque had a much larger collagen fiber content than the group of model (Figures 1C, D, *p* < 0.05). These findings illustrate that TIIA can reduce the relative lumen area of lipid-rich plaques, enhance plaque stability, and thus inhibit aortic plaque progression in mice.

### 3.2 TIIA improves blood lipid levels in mice

**FIGURE 2:**
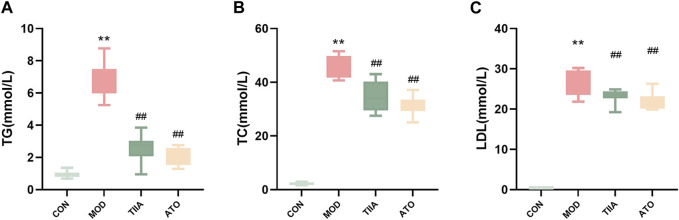
Serum lipid profiles of LDLR^−/−^ mice (n = 8). **(A)** Serum TC change of mice. **(B)** Serum TG change of mice. **(C)** Serum LDL change of mice. CON, MOD, ATO, and TIIA represent the control group, model group, atorvastatin group, and tanshinone IIA group, respectively. **p < 0.01 vs. CON; ##*p* < 0.01 vs. MOD.

Elevated blood lipid levels are known to increase the risk of cardiovascular disease (Ginsberg et al., 2021). To test the impact of TIIA on blood lipids, we analyzed the serum levels of TC, TG, and LDL in different groups of mice. The model mice had the worst lipid metabolism and the highest blood lipid levels (TC, TG, and LDL). Both TIIA and ATO enhanced lipid metabolism and reduced blood lipid levels (Figures 2A–C, *p* < 0.01). It can be demonstrated that TIIA could decrease blood lipids and mitigate the negative effects of AS risk factors, which is advantageous in the treatment of atherosclerosis.

### 3.3 TIIA enhances the expression of efferocytosis-related signaling (CX3CR1) and clearance of apoptotic cells in mouse aorta

**FIGURE 3:**
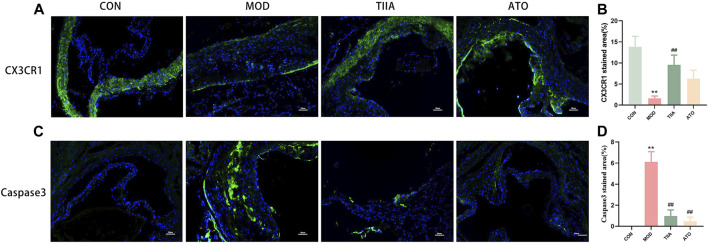
Immunofluorescence stain of the aortic sinus (n = 3). **(A,B)** CX3CR1 protein in the adjacent area of the aortic sinus for each group of mice and the positive areas of the aortic sinus were quantitated. **(C,D**) Caspase3 Podoplanin in the adjacent area of the aortic sinus for each group of mouse, and the positive areas of the whole aorta was quantitated adjacent area of the aortic sinus. CON, MOD, ATO, and TIIA represent the control group, model group, atorvastatin group, and tanshinone IIA group, respectively. **p < 0.01 vs. CON; ##*p* < 0.01 vs. MOD.

The efficient efferocytosis can effectively remove necrotic cells from the lesion and reduce AS. To analyze the effect of TIIA on the efferocytosis-related signal and the apoptotic cell clearance ability in each group, we detected the efferocytosis-related signal and apoptotic cells in the aorta of each group by immunofluorescence. Our research indicated that the efferocytosis-related signal (CX3CR1) was lowest in the MOD (*p* < 0.01), and there was an obvious increase in apoptotic cells in the aorta of the model mice (*p* < 0.01). No apoptotic cells were detected in the control group. In contradistinction, after TIIA intervention, the efferocytosis-related signal in the aorta of the mice was enhanced and the apoptotic cells were noticeably reduced compared to the model group. (Figures 3A–D, *p* < 0.01). These findings imply that TIIA may enhance the clearance of apoptotic cells at the lesion and improve AS via promoting efferocytosis. The findings of this study indicate that TIIA has the potential to improve efferocytosis, which can improve the clearance of apoptotic cells from the site of injury and ultimately improve AS.

### 3.4 TIIA promotes the expression of efferocytosis-related molecules in the mouse aorta and RAW264.7 cells

**FIGURE 4:**
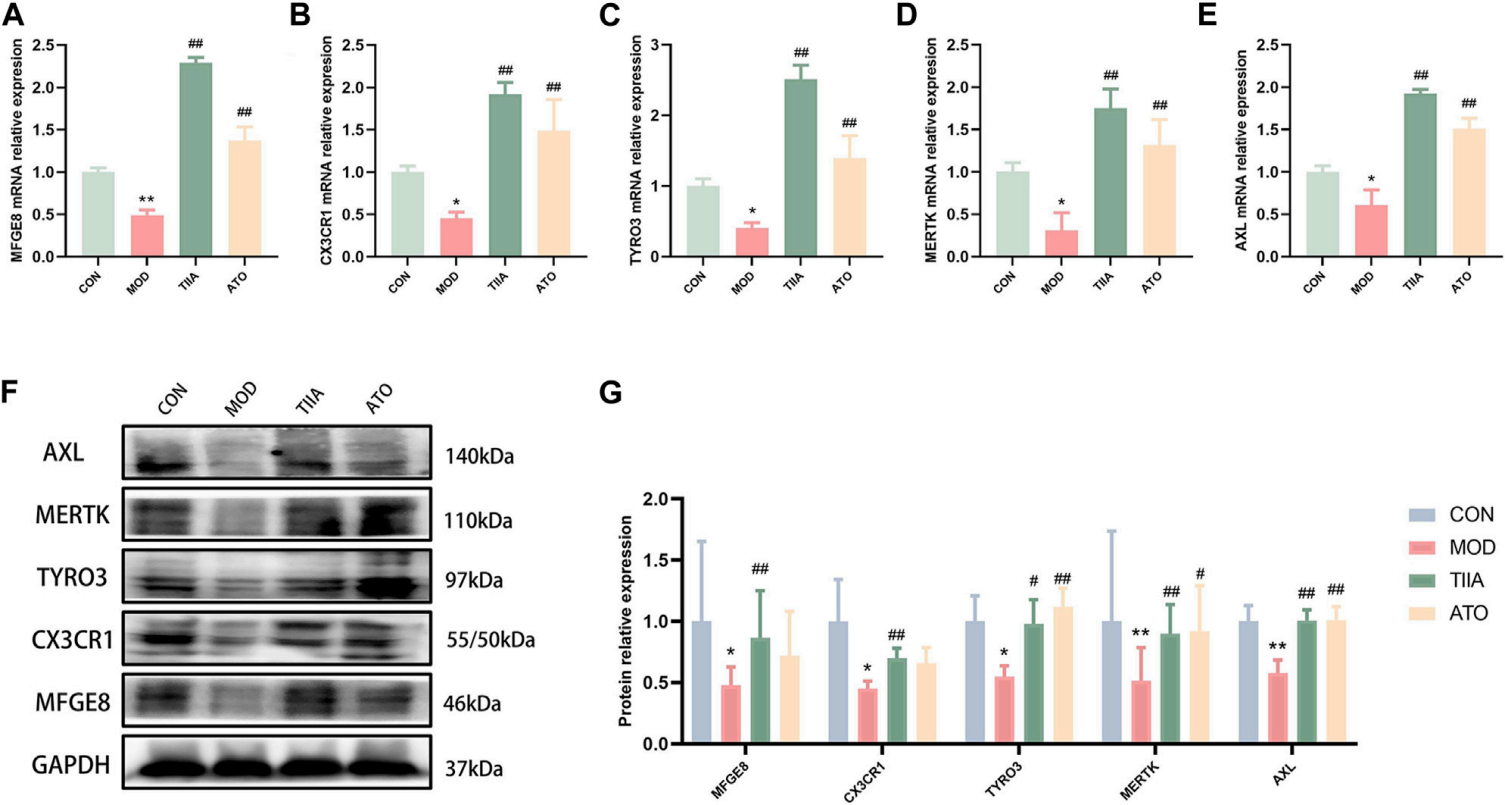
The effect of TIIA on efferocytosis-related molecule expression in LDLR^−/−^ mice (n = 3). **(A-E)** RT-qPCR was used to detect MFGE8, CX3CR1, MERTK, AXL, and TYRO3 gene expression in the aorta. **(F-G)** The protein expression of MFGE8, CX3CR1, MERTK, AXL, and TYRO3 in the aorta was examined using a Western blotting technique, and the quantitative results were shown. CON, MOD, ATO, and TIIA represent the control group, model group, atorvastatin group, and tanshinone IIA group, respectively. *p < 0.05 vs. CON; **p < 0.01 vs. CON; #*p* < 0.05, ##*p* < 0.01 vs. MOD.

**FIGURE 5:**
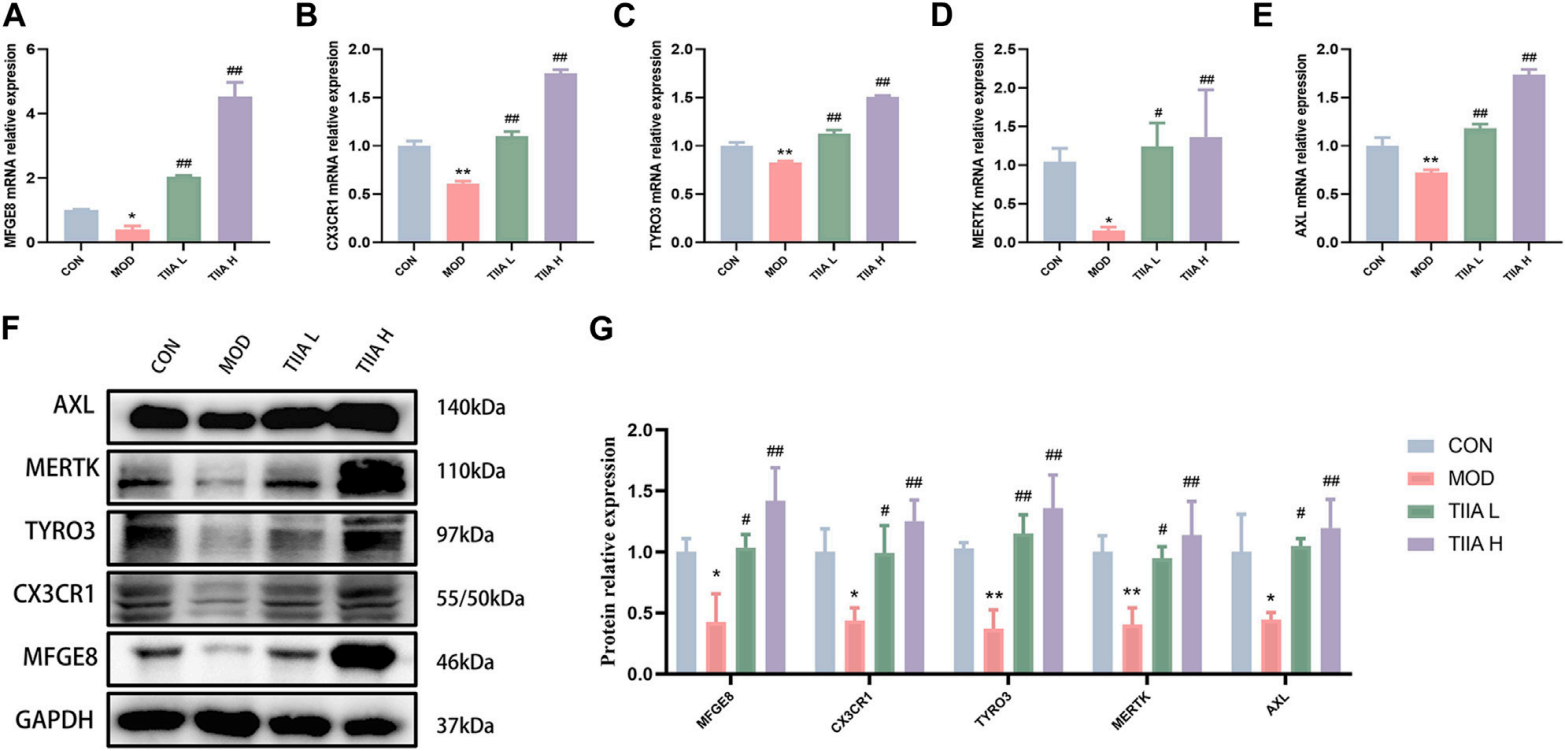
Effect of TIIA on the expression of efferocytosis-related molecules in RAW264.7 cells (n = 3). **(A-E)** RT-qPCR was used to evaluate the gene expression of MFGE8, CX3CR1, MERTK, AXL, and TYRO3.**(F-G)** Western blot was used to quantify the expression of MFGE8, CX3CR1, MERTK, AXL, and TYRO3 proteins in each group. CON, control group; MOD, model group (with ox-LDL 50 ug/mL); TIIA L, low Tanshinone IIA group (with ox-LDL 50 ug/mL + TIIA 20 uM/L); TIIA H, high Tanshinone IIA group (with ox-LDL 50 ug/mL + TIIA 40 uM/L). *p < 0.05 vs. CON, **p < 0.01 vs. CON; #*p* < 0.05, ##*p* < 0.01 vs. MOD.

To further identify the molecular biological mechanism of TIIA-regulated efferocytosis, we examined the expression of efferocytosis-related signals in mouse aorta by RT-qPCR and Western blot. The RT-qPCR results showed a significant decrease in mRNA levels of MERTK, CX3CR1, MFGE8, AXL, and TYRO3 in the model mice (*p* < 0.01 or *p* < 0.05); TIIA intervention effectively reversed this trend (Figures 4A–E, *p* < 0.01). Based on the Western blot results, the expression levels of MERTK, CX3CR1, MFGE8, AXL, and TYRO3 proteins were in agreement with their respective mRNA levels (Figures 4F, G). The trend of these molecules in RAW264.7 cells was the same as in the mouse aorta (Figures 5A–G).

### 3.5 TIIA attenuates the macrophage-derived foam cell accumulation

**FIGURE 6:**
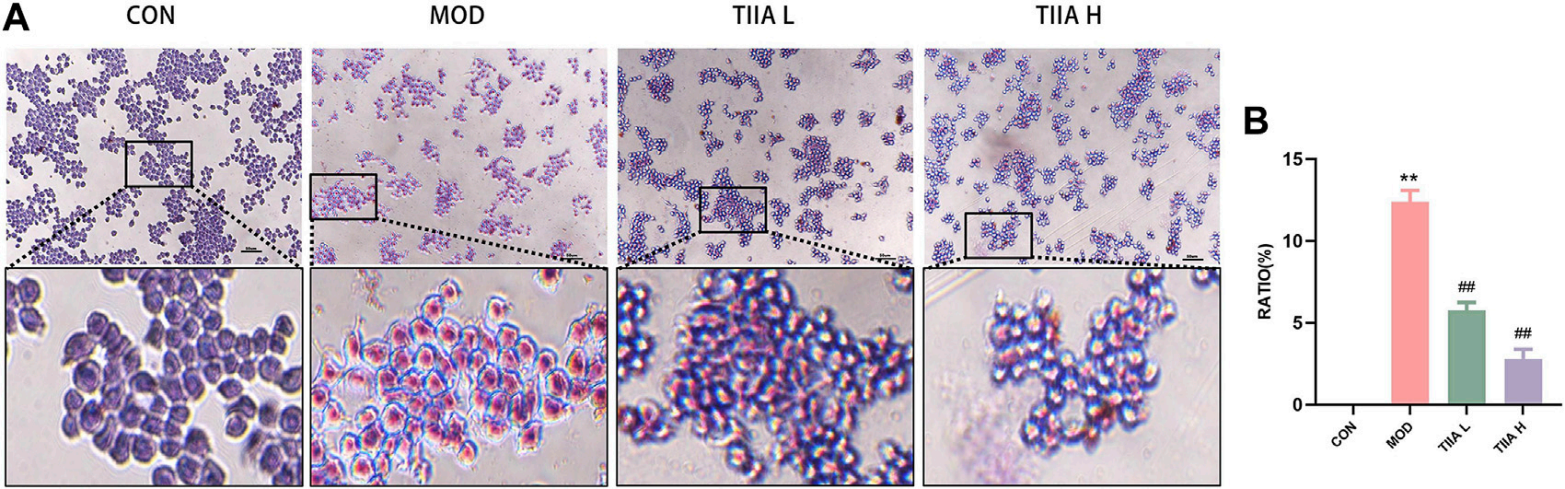
Effect of TIIA on macrophage-derived foam cells in RAW264.7 cells (n = 3). **(A)** Representative images of ORO staining in RAW264.7 cells. **(B)** The corresponding quantitative analysis for each group. CON, control group; MOD, model group (with ox-LDL 50 ug/mL); TIIA L, low Tanshinone IIA group (with ox-LDL 50 ug/mL + TIIA 20 uM/L); TIIA H, high Tanshinone IIA group (with ox-LDL 50 ug/mL + TIIA 40 uM/L). **p < 0.01 vs. CON; ##*p* < 0.01 vs. MOD.

Macrophages, the most critical cell type in AS, are deeply involved in the process of atherogenesis and the development of AS. Macrophage-derived foam cell accumulation is a fundamental cause of atherosclerotic plaque formation (Zheng et al., 2021). In this study, an *in vitro* foam cell model was developed using RAW264.7 cells to investigate the impact of TIIA on the development of macrophage-derived foam cells. The accumulation of macrophage-derived foam cells was evaluated through ORO staining after TIIA intervention. The results in Figures 6A, B indicate that TIIA significantly decreased the accumulation of macrophage-derived foam cells (*p* < 0.01).

### 3.6 TIIA enhances the expression of efferocytosis-related signal (CX3CR1) and improves efferocytosis in RAW264.7 cells

**FIGURE 7:**
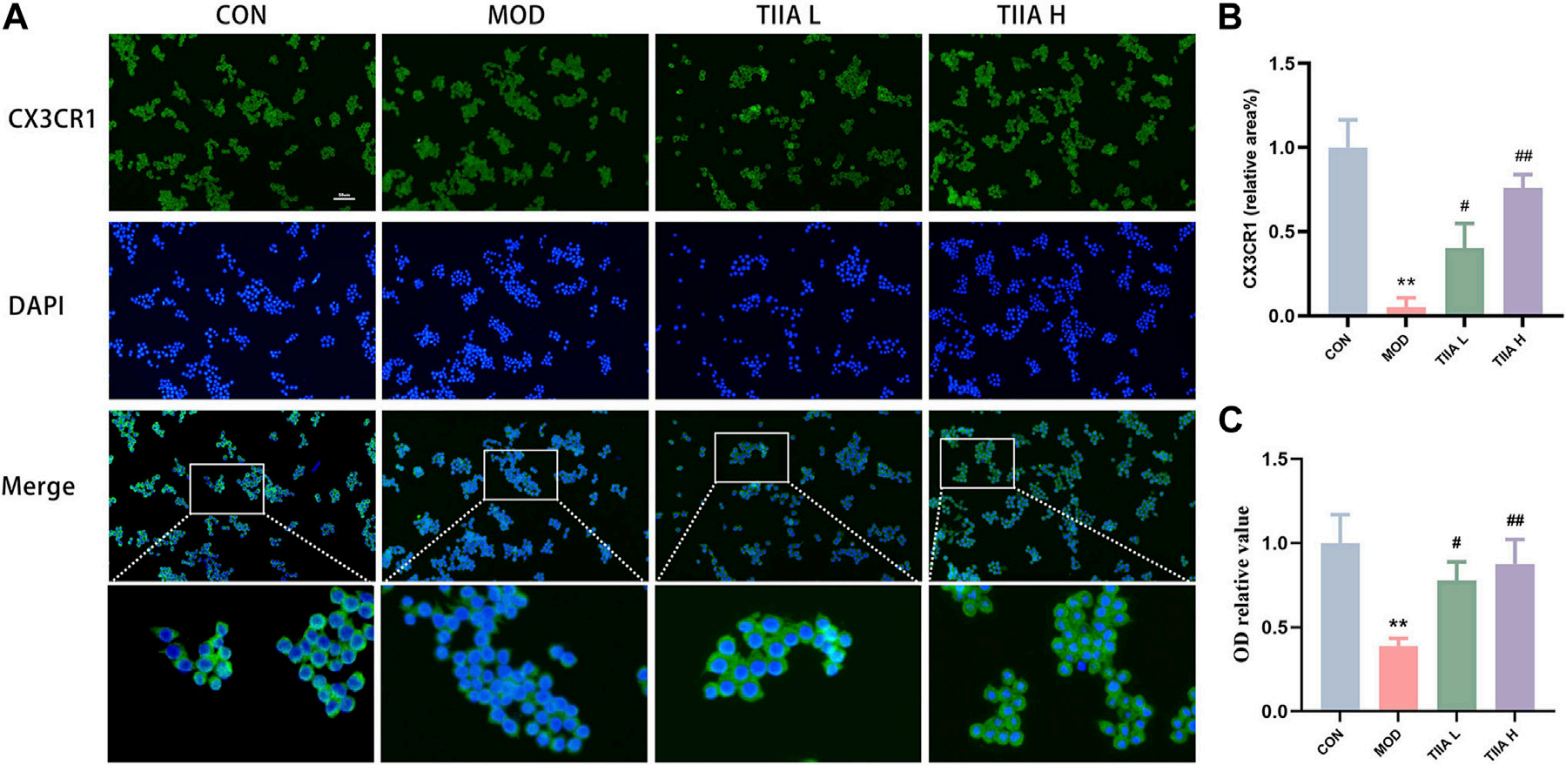
Effect of TIIA on efferocytosis-related signal and efferocytosis rate in RAW264.7 cells. **(A,B)** Immunofluorescence staining was performed to show CX3CR1 in RAW264.7 cells, and corresponding quantitative analysis was shown. **(C)** The neutral red uptake test was used to assess the effects of TIIA on the phagocytic capability of RAW264.7 cells. CON, control group; MOD, model group (with ox-LDL 50 ug/mL); TIIA L, low Tanshinone IIA group (with ox-LDL 50 ug/mL + TIIA 20 uM/L); TIIA H, high Tanshinone IIA group (with ox-LDL 50 ug/mL + TIIA 40 uM/L). *p < 0.05 vs. CON; #*p* < 0.05, ##*p* < 0.01 vs. MOD.

The efficient efferocytosis effectively degrades lipids phagocytosed by macrophages, lessens the generation of foam cells derived from macrophages, and improves AS finally. Therefore, we examined the impact of TIIA on the expression of CX3CR1 (efferocytosis-related molecule) and the phagocytic ability in RAW264.7 cells. The results presented in Figures 7A, B demonstrate that the expression of CX3CR1 was notably weaker in the model group than in the control group (*p* < 0.01). However, after TIIA intervention, the expression of CX3CR1 was significantly enhanced (*p* < 0.05 or *p* < 0.01). Additionally, the neutral red uptake assay indicated that TIIA effectively increased the efferocytosis rate of RAW264.7 cells (Figure 7C). The above results indicated that TIIA enhanced the expression of efferocytosis-related signal (CX3CR1) in RAW264.7 cells and increased the efferocytosis rate of RAW264.7 cells.

## 4 Discussion

Studies have shown that knockdown or silencing of LDLR affects cholesterol transport and disrupts intracellular cholesterol homeostasis, causing cholesterol to accumulate in the arterial wall and inducing AS (Gomes et al., 2022). Our study found that TIIA reduced the area of aortic lipid-rich plaques and the risk of plaque rupture and detachment in western diet-induced LDLR^−/−^ mice and improved AS. Based on our research studying the mechanisms *in vivo* and *in vitro*, we discovered the potential therapeutic mechanism of TIIA for treating AS might be that TIIA improves macrophage clearance of apoptotic cells and excess lipids via promoting macrophage efferocytosis. Previous studies have demonstrated the positive impact of TIIA on various diseases such as cardiovascular disease, diabetes, obesity, and cancer (Guo et al., 2020; Ansari et al., 2021). For example, TIIA may alleviate AS by influencing gut microbiota and cholesterol metabolism (Jia et al., 2016; Yang et al., 2021). In addition, TIIA may play a role in regulating oxidative stress and endothelial cell function (Lu et al., 2022), endoplasmic reticulum stress and endoplasmic reticulum stress (Li et al., 2022), iron death (Ni et al., 2022), macrophage polarization (Zhao et al., 2022), and cell cycle functions (Zhang et al., 2022). However, the effect of TIIA on macrophage efferocytosis has yet to receive much attention. Our research findings have provided experimental evidence in the laboratory to support novel therapeutic targets for AS treatment to a certain extent.

Macrophages are heterogeneous cells, and different functions and phenotypes of macrophages have different roles in disease (Artyomov et al., 2016). In AS, when macrophages engulf excessive lipids that cannot be removed promptly, they develop macrophage-derived foam cells, a hallmark of plaque formation and development (Yuan et al., 2012; Duan et al., 2022). Efferocytosis is the process by which phagocytes (mainly macrophages) engulf and digest apoptotic cargo *in vivo*, essential for maintaining homeostasis and damage repair *in vivo* (Mehrotra and Ravichandran, 2022). Therefore, in the present study, we selected ox-LDL-induced RAW264.7 cells *in vitro* to further explore the mechanism of TIIA treatment of AS.

Efficient efferocytosis facilitates the healing of tissue damage caused by diabetes (Maschalidi et al., 2022) and the regression of inflammation in inflammatory diseases (Ampomah et al., 2022; Ge et al., 2022; Glinton et al., 2022). Our study showed that TIIA effectively increased lipid clearance by RAW264.7 cells and reduced macrophage-derived foam cells. The mechanism might be related to the increased efferocytosis of TIIA on macrophages. Previous studies have found that chronic nutrient overload impairs efferocytosis and causes the accumulation of apoptotic cells *in vivo*, which is consistent with our study (Wang et al., 2023). “Apoptotic cell (AC) finding,” “AC binding,” and “AC degradation” are the three steps of efferocytosis (Doran et al., 2020). Each phase relies on the corresponding efferocytosis-related signals to function. CX3CR1 is a significant signal in the “AC finding” phase and plays a vital role in treating neuroinflammation and chronic kidney disease (Cormican and Griffin, 2021; Subbarayan et al., 2022). Shweta S Puntambekar et al. found that CX3CR1-deficient mice exhibit a “degenerative” phenotype. The mechanism is related to impaired microglia function leading to increased oxidative stress and over-activation of pro-inflammatory signals (Puntambekar et al., 2022). Microglia, a resident macrophage-like population in the central nervous system (CNS), share many structural and functional similarities with macrophages that reside elsewhere (Nayak et al., 2014; Prinz et al., 2019; Borst et al., 2021). Our *ex vivo* studies revealed that CX3CR1 expression was downregulated in the AS model group in both the immune signaling and transcriptional levels, and TIIA reversed this downward trend. In addition, the modulator MFGE8, one of the cytosolic burial signals, promotes enterocyte triglyceride hydrolase activity, reduces postprandial lipids, decreases lipid accumulation in enterocytes, and ameliorates hepatic steatosis and inflammation (Khalifeh-Soltani et al., 2016; Zhang et al., 2021). In this study, TIIA upregulated MFGE8 expression, promoted efferocytosis, enhanced lipid phagocytosis and degradation by macrophages, reduced macrophage-derived foam cell formation, and improved AS. In addition, TYRO3, MERTK, and AXL showed the same trend as CX3CR1 and MFGE8. In summary, we think TIIA could promote efferocytosis and improve AS by upregulating efferocytosis-related signals.

Notably, ox-LDL can compete with apoptotic cells to bind efferocytosis-related signals as AS progresses. In response to this situation, macrophages exhibit a stronger phagocytic capacity to compensate for this demand, which may lead to macrophage overload, further impairing efferocytosis and promoting the progression of AS. Our finding is that TIIA promotes the phagocytosis of harmful substances by macrophages and, more importantly, the degradation of harmful substances by macrophages. TIIA promotes efferocytosis and timely degradation and removal of these harmful substances so that macrophages can maintain their functions efficiently and effectively and ultimately achieve the purpose of treating AS. Our pathological results showed that both apoptotic cells (Figures 3A–D) and lipid accumulation (Figures 6A, B) at the lesion were significantly reduced after TIIA intervention, providing evidence for Our views. Although we have not yet clarified the specific mechanism by which TIIA promotes the ability of macrophages to have more effective digestion and degradation of harmful substances, this also provides a direction for us to continue our research.

**FIGURE 8:**
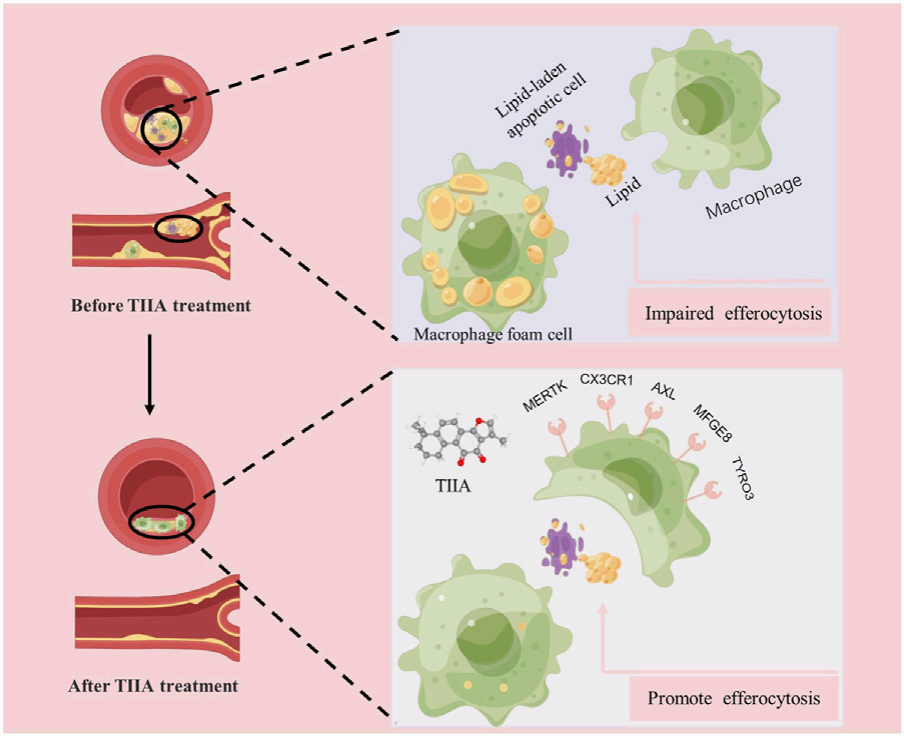
Mechanisms of TIIA in treating AS.

In conclusion, our evidence jointly supports that TIIA may be therapeutic in AS by enhancing efferocytosis in macrophages during AS lesions (Figure 8). In the future, continued in-depth study of the specific mechanism by which TIIA enables macrophages to maintain strong phagocytosis and digestion and absorption in AS may advance the progress of TIIA as a target therapeutic agent for macrophage cytosolic action.

## Data Availability

The original contributions presented in the study are included in the article/Supplementary Materials, further inquiries can be directed to the corresponding author.

## References

[B1] Ampomah P. B. Cai B. Sukka S. R. Gerlach B. D. Yurdagul A. Jr. Wang X. (2022). Macrophages use apoptotic cell-derived methionine and DNMT3A during efferocytosis to promote tissue resolution. Nat. Metab. 4 (4), 444–457. 10.1038/s42255-022-00551-7 35361955 PMC9050866

[B2] Ansari M. A. Khan F. B. Safdari H. A. Almatroudi A. Alzohairy M. A. Safdari M. (2021). Prospective therapeutic potential of Tanshinone IIA: An updated overview. Pharmacol. Res. 164, 105364. 10.1016/j.phrs.2020.105364 33285229

[B3] Artyomov M. N. Sergushichev A. Schilling J. D. (2016). Integrating immunometabolism and macrophage diversity. Semin. Immunol. 28 (5), 417–424. 10.1016/j.smim.2016.10.004 27771140 PMC5333784

[B4] Badjatiya A. Rao S. V. (2019). Advances in antiplatelet and anticoagulant therapies for NSTE-ACS. Curr. Cardiol. Rep. 21 (1), 3. 10.1007/s11886-019-1090-3 30637536

[B5] Björkegren J. L. M. Lusis A. J. (2022). Atherosclerosis: Recent developments. Cell 185 (10), 1630–1645. 10.1016/j.cell.2022.04.004 35504280 PMC9119695

[B6] Borst K. Dumas A. A. Prinz M. (2021). Microglia: Immune and non-immune functions. Immunity 54 (10), 2194–2208. 10.1016/j.immuni.2021.09.014 34644556

[B7] Brown D. G. Wobst H. J. (2021). A decade of FDA-approved drugs (2010-2019): Trends and future directions. J. Med. Chem. 64 (5), 2312–2338. 10.1021/acs.jmedchem.0c01516 33617254

[B8] Chen W. Schilperoort M. Cao Y. Shi J. Tabas I. Tao W. (2022). Macrophage-targeted nanomedicine for the diagnosis and treatment of atherosclerosis. Nat. Rev. Cardiol. 19 (4), 228–249. 10.1038/s41569-021-00629-x 34759324 PMC8580169

[B9] Cormican S. Griffin M. D. (2021). Fractalkine (CX3CL1) and its receptor CX3CR1: A promising therapeutic target in chronic kidney disease? Front. Immunol. 12, 664202. 10.3389/fimmu.2021.664202 34163473 PMC8215706

[B10] Doddapattar P. Dev R. Ghatge M. Patel R. B. Jain M. Dhanesha N. (2022). Myeloid cell PKM2 deletion enhances efferocytosis and reduces atherosclerosis. Circ. Res. 130 (9), 1289–1305. 10.1161/circresaha.121.320704 35400205 PMC9050913

[B11] Doran A. C. Yurdagul A. Jr. Tabas I. (2020). Efferocytosis in health and disease. Nat. Rev. Immunol. 20 (4), 254–267. 10.1038/s41577-019-0240-6 31822793 PMC7667664

[B12] Duan Y. Zhang X. Zhang X. Lin J. Shu X. Man W. (2022). Inhibition of macrophage-derived foam cells by Adipsin attenuates progression of atherosclerosis. Biochim. Biophys. Acta Mol. Basis Dis. 1868 (12), 166533. 10.1016/j.bbadis.2022.166533 36064133

[B13] Ge Y. Huang M. Yao Y. M. (2022). Efferocytosis and its role in inflammatory disorders. Front. Cell Dev. Biol. 10, 839248. 10.3389/fcell.2022.839248 35281078 PMC8913510

[B14] Gerlach B. D. Ampomah P. B. Yurdagul A. Jr. Liu C. Lauring M. C. Wang X. (2021). Efferocytosis induces macrophage proliferation to help resolve tissue injury. Cell Metab. 33 (12), 2445–2463.e8. 10.1016/j.cmet.2021.10.015 34784501 PMC8665147

[B15] Ginsberg H. N. Packard C. J. Chapman M. J. Borén J. Aguilar-Salinas C. A. Averna M. (2021). Triglyceride-rich lipoproteins and their remnants: Metabolic insights, role in atherosclerotic cardiovascular disease, and emerging therapeutic strategies-a consensus statement from the European atherosclerosis society. Eur. Heart J. 42 (47), 4791–4806. 10.1093/eurheartj/ehab551 34472586 PMC8670783

[B16] Gisterå A. Ketelhuth D. F. J. Malin S. G. Hansson G. K. (2022). Animal models of atherosclerosis-supportive notes and tricks of the trade. Circ. Res. 130 (12), 1869–1887. 10.1161/circresaha.122.320263 35679358

[B17] Glinton K. E. Ma W. Lantz C. Grigoryeva L. S. DeBerge M. Liu X. (2022). Macrophage-produced VEGFC is induced by efferocytosis to ameliorate cardiac injury and inflammation. J. Clin. Invest. 132 (9), e140685. 10.1172/jci140685 35271504 PMC9057589

[B18] Gomes D. Wang S. Goodspeed L. Turk K. E. Wietecha T. Liu Y. (2022). Comparison between genetic and pharmaceutical disruption of Ldlr expression for the development of atherosclerosis. J. Lipid Res. 63 (3), 100174. 10.1016/j.jlr.2022.100174 35101425 PMC8953673

[B19] Guo R. Li L. Su J. Li S. Duncan S. E. Liu Z. (2020). Pharmacological activity and mechanism of tanshinone IIA in related diseases. Drug Des. Devel Ther. 14, 4735–4748. 10.2147/dddt.S266911 PMC7653026 33192051

[B20] Hao P. Jiang F. Cheng J. Ma L. Zhang Y. Zhao Y. (2017). Traditional Chinese medicine for cardiovascular disease: Evidence and potential mechanisms. J. Am. Coll. Cardiol. 69 (24), 2952–2966. 10.1016/j.jacc.2017.04.041 28619197

[B21] Jia D. Chen S. Bai P. Luo C. Liu J. Sun A. (2022). Cardiac resident macrophage-derived legumain improves cardiac repair by promoting clearance and degradation of apoptotic cardiomyocytes after myocardial infarction. Circulation 145 (20), 1542–1556. 10.1161/circulationaha.121.057549 35430895

[B22] Jia L. Q. Zhang N. Xu Y. Chen W. N. Zhu M. L. Song N. (2016). Tanshinone IIA affects the HDL subfractions distribution not serum lipid levels: Involving in intake and efflux of cholesterol. Arch. Biochem. Biophys. 592, 50–59. 10.1016/j.abb.2016.01.001 26820219

[B23] Jin Y. Liu Y. Xu L. Xu J. Xiong Y. Peng Y. (2022). Novel role for caspase 1 inhibitor VX765 in suppressing NLRP3 inflammasome assembly and atherosclerosis via promoting mitophagy and efferocytosis. Cell Death Dis. 13 (5), 512. 10.1038/s41419-022-04966-8 35641492 PMC9156694

[B24] Kasikara C. Schilperoort M. Gerlach B. Xue C. Wang X. Zheng Z. (2021). Deficiency of macrophage PHACTR1 impairs efferocytosis and promotes atherosclerotic plaque necrosis. J. Clin. Invest. 131 (8), e145275. 10.1172/jci145275 33630758 PMC8262505

[B25] Khalifeh-Soltani A. Gupta D. Ha A. Iqbal J. Hussain M. Podolsky M. J. (2016). Mfge8 regulates enterocyte lipid storage by promoting enterocyte triglyceride hydrolase activity. JCI Insight 1 (18), e87418. 10.1172/jci.insight.87418 27812539 PMC5085605

[B26] Li Y. Fu Y. Sun J. Shen J. Liu F. Ning B. (2022). Tanshinone IIA alleviates NLRP3 inflammasome-mediated pyroptosis in Mycobacterium tuberculosis-(H37Ra-) infected macrophages by inhibiting endoplasmic reticulum stress. J. Ethnopharmacol. 282, 114595. 10.1016/j.jep.2021.114595 34517060

[B27] Libby P. (2021). The changing landscape of atherosclerosis. Nature 592 (7855), 524–533. 10.1038/s41586-021-03392-8 33883728

[B28] Lu T. C. Wu Y. H. Chen W. Y. Hung Y. C. (2022). Targeting oxidative stress and endothelial dysfunction using tanshinone IIA for the treatment of tissue inflammation and fibrosis. Oxid. Med. Cell Longev. 2022, 2811789. 10.1155/2022/2811789 35432718 PMC9010204

[B29] Manta C. P. Leibing T. Friedrich M. Nolte H. Adrian M. Schledzewski K. (2022). Targeting of scavenger receptors stabilin-1 and stabilin-2 ameliorates atherosclerosis by a plasma proteome switch mediating monocyte/macrophage suppression. Circulation 146 (23), 1783–1799. 10.1161/circulationaha.121.058615 36325910

[B30] Maschalidi S. Mehrotra P. Keçeli B. N. De Cleene H. K. L. Lecomte K. Van der Cruyssen R. (2022). Targeting SLC7A11 improves efferocytosis by dendritic cells and wound healing in diabetes. Nature 606 (7915), 776–784. 10.1038/s41586-022-04754-6 35614212

[B31] Mehrotra P. Ravichandran K. S. (2022). Drugging the efferocytosis process: Concepts and opportunities. Nat. Rev. Drug Discov. 21 (8), 601–620. 10.1038/s41573-022-00470-y 35650427 PMC9157040

[B32] Nayak D. Roth T. L. McGavern D. B. (2014). Microglia development and function. Annu. Rev. Immunol. 32, 367–402. 10.1146/annurev-immunol-032713-120240 24471431 PMC5001846

[B33] Ni H. Ruan G. Sun C. Yang X. Miao Z. Li J. (2022). Tanshinone IIA inhibits gastric cancer cell stemness through inducing ferroptosis. Environ. Toxicol. 37 (2), 192–200. 10.1002/tox.23388 34661962

[B34] Pierno S. Musumeci O. (2023). Pharmacotherapy of the lipid-lowering drugs: Update on efficacy and risk. Int. J. Mol. Sci. 24 (2), 996. 10.3390/ijms24020996 36674512 PMC9864443

[B35] Prinz M. Jung S. Priller J. (2019). Microglia biology: One century of evolving concepts. Cell 179 (2), 292–311. 10.1016/j.cell.2019.08.053 31585077

[B36] Puntambekar S. S. Moutinho M. Lin P. B. Jadhav V. Tumbleson-Brink D. Balaji A. (2022). CX3CR1 deficiency aggravates amyloid driven neuronal pathology and cognitive decline in Alzheimer's disease. Mol. Neurodegener. 17 (1), 47. 10.1186/s13024-022-00545-9 35764973 PMC9241248

[B37] Sherwood M. W. Cyr D. D. Jones W. S. Becker R. C. Berkowitz S. D. Washam J. B. (2016). Use of dual antiplatelet therapy and patient outcomes in those undergoing percutaneous coronary intervention: The ROCKET AF trial. JACC Cardiovasc Interv. 9 (16), 1694–1702. 10.1016/j.jcin.2016.05.039 27539689 PMC6921699

[B38] Simion V. Zhou H. Haemmig S. Pierce J. B. Mendes S. Tesmenitsky Y. (2020). A macrophage-specific lncRNA regulates apoptosis and atherosclerosis by tethering HuR in the nucleus. Nat. Commun. 11 (1), 6135. 10.1038/s41467-020-19664-2 33262333 PMC7708640

[B39] Subbarayan M. S. Joly-Amado A. Bickford P. C. Nash K. R. (2022). CX3CL1/CX3CR1 signaling targets for the treatment of neurodegenerative diseases. Pharmacol. Ther. 231, 107989. 10.1016/j.pharmthera.2021.107989 34492237

[B40] Tsao C. W. Aday A. W. Almarzooq Z. I. Alonso A. Beaton A. Z. Bittencourt M. S. (2022). Heart disease and stroke statistics-2022 update: A report from the American heart association. Circulation 145 (8), e153–e639. 10.1161/cir.0000000000001052 35078371

[B41] Virmani R. Burke A. P. Farb A. Kolodgie F. D. (2006). Pathology of the vulnerable plaque. J. Am. Coll. Cardiol. 47 (8), C13–C18. 10.1016/j.jacc.2005.10.065 16631505

[B42] Wang N. Zhang X. Ma Z. Niu J. Ma S. Wenjie W. (2020). Combination of tanshinone IIA and astragaloside IV attenuate atherosclerotic plaque vulnerability in ApoE(-/-) mice by activating PI3K/AKT signaling and suppressing TRL4/NF-κB signaling. Biomed. Pharmacother. 123, 109729. 10.1016/j.biopha.2019.109729 31887543

[B43] Wang X. He Q. Zhou C. Xu Y. Liu D. Fujiwara N. (2023). Prolonged hypernutrition impairs TREM2-dependent efferocytosis to license chronic liver inflammation and NASH development. Immunity 56 (1), 58–77.e11. 10.1016/j.immuni.2022.11.013 36521495 PMC9839616

[B44] Wen J. Chang Y. Huo S. Li W. Huang H. Gao Y. (2020). Tanshinone IIA attenuates atherosclerosis via inhibiting NLRP3 inflammasome activation. Aging (Albany NY) 13 (1), 910–932. 10.18632/aging.202202 33290264 PMC7835056

[B45] Yang R. Yin D. Yang D. Liu X. Zhou Q. Pan Y. (2021). Xinnaokang improves cecal microbiota and lipid metabolism to target atherosclerosis. Lett. Appl. Microbiol. 73 (6), 779–792. 10.1111/lam.13573 34596907

[B46] Yuan Y. Li P. Ye J. (2012). Lipid homeostasis and the formation of macrophage-derived foam cells in atherosclerosis. Protein Cell 3 (3), 173–181. 10.1007/s13238-012-2025-6 22447659 PMC4875426

[B47] Zhang L. Tian R. Yao X. Zhang X. J. Zhang P. Huang Y. (2021). Milk fat globule-epidermal growth factor-factor 8 improves hepatic steatosis and inflammation. Hepatology 73 (2), 586–605. 10.1002/hep.31277 32297339

[B48] Zhang W. Liu C. Li J. Lu Y. Li H. Zhuang J. (2022). Tanshinone IIA: New perspective on the anti-tumor mechanism of A traditional natural medicine. Am. J. Chin. Med. 50 (1), 209–239. 10.1142/s0192415x22500070 34983327

[B49] Zhao J. Y. Pu J. Fan J. Feng X. Y. Xu J. W. Zhang R. (2022). Tanshinone IIA prevents acute lung injury by regulating macrophage polarization. J. Integr. Med. 20 (3), 274–280. 10.1016/j.joim.2022.01.006 35181255

[B50] Zheng S. Huang H. Li Y. Wang Y. Zheng Y. Liang J. (2021). Yin-xing-tong-mai decoction attenuates atherosclerosis via activating PPARγ-LXRα-ABCA1/ABCG1 pathway. Pharmacol. Res. 169, 105639. 10.1016/j.phrs.2021.105639 33932607

