# Supplementary material for: Tanshinone IIA alleviates atherosclerosis in LDLR−/− mice by regulating efferocytosis of macrophages
Source: Front Pharmacol. 2023 Oct 11;14:1233709. doi: 10.3389/fphar.2023.1233709 (PMC10598641; doi:10.3389/fphar.2023.1233709)
Supplement: Supplementary file 1 [file Table1.docx]

The supplementary material for this article can be found online at <https://www.jianguoyun.com/p/Ddxqt-sQ15eBDBiBlpwFIAA>

and <https://www.jianguoyun.com/p/DVVaIZsQ15eBDBiHlpwFIAA>.

.
